# Longitudinal study of neuropathy, microangiopathy, and autophagy in sural nerve: Implications for diabetic neuropathy

**DOI:** 10.1002/brb3.763

**Published:** 2017-07-12

**Authors:** Simin Mohseni, Medeea Badii, Axel Kylhammar, Niels O. B. Thomsen, Karl‐Fredrik Eriksson, Rayaz A. Malik, Ingmar Rosén, Lars B. Dahlin

**Affiliations:** ^1^ Department of Clinical and Experimental Medicine Division of Cell Biology Linköping University Linköping Sweden; ^2^ Department of Hand Surgery Skåne University Hospital Malmö Sweden; ^3^ Vascular Department of Angiology Skåne University Hospital Malmö Sweden; ^4^ Weill Cornell Medicine‐Qatar Qatar Foundation Doha Qatar; ^5^ Division of Cardiovascular Sciences Manchester Academic Health Science Centre Central Manchester University Hospitals NHS Foundation Trust Manchester UK; ^6^ Department of Neurophysiology Skåne University Hospital Lund Sweden; ^7^ Department of Translational Medicine – Hand Surgery Lund University Malmö Sweden

**Keywords:** autophagy, diabetes, endoneurial capillary, impaired glucose tolerance, morphometry, peripheral neuropathy

## Abstract

**Objectives:**

The progression and pathophysiology of neuropathy in impaired glucose tolerance (IGT) and type 2 diabetes (T2DM) is poorly understood, especially in relation to autophagy. This study was designed to assess whether the presence of autophagy‐related structures was associated with sural nerve fiber pathology, and to investigate if endoneurial capillary pathology could predict the development of T2DM and neuropathy.

**Patients and Methods:**

Sural nerve physiology and ultrastructural morphology were studied at baseline and 11 years later in subjects with normal glucose tolerance (NGT), IGT, and T2DM.

**Results:**

Subjects with T2DM had significantly lower sural nerve amplitude compared to subjects with NGT and IGT at baseline. Myelinated and unmyelinated fiber, endoneurial capillary morphology, and the presence and distribution of autophagy structures were comparable between groups at baseline, except for a smaller myelinated axon diameter in subjects with T2DM and IGT compared to NGT. The baseline values of the subjects with NGT and IGT who converted to T2DM 11 years later demonstrated healthy smaller endoneurial capillary and higher *g*‐ratio versus subjects who remained NGT. At follow‐up, T2DM showed a reduction in nerve conduction, amplitude, myelinated fiber density, unmyelinated axon diameter, and autophagy structures in myelinated axons. Endothelial cell area and total diffusion barrier was increased versus baseline.

**Conclusions:**

We conclude that small healthy endoneurial capillary may presage the development of T2DM and neuropathy. Autophagy occurs in human sural nerves and can be affected by T2DM. Further studies are warranted to understand the role of autophagy in diabetic neuropathy.

## 1 INTRODUCTION

Diabetic neuropathy can affect 50% of patients with diabetes and is associated with increased morbidity and mortality (Wild, Roglic, Green, Sicree, & King, 2004). There is no general consensus regarding the pathogenesis of the diabetic neuropathy (Callaghan, Hur, & Feldman, 2012) and currently, there is no FDA approved therapy (Malik, 2016). An array of factors, such as dysfunction of mitochondria and endoplasmic reticulum, hypercholesterolemia, endothelial dysfunction, and ischemia/hypoxia, are considered to play an important role in the development of diabetic neuropathy (Callaghan et al., 2012; Cashman & Höke, 2015; Feldman, Nave, Jensen, & Bennett, 2017; Gonçalves et al., 2017). Pathological studies of sural nerve biopsies from patients with diabetic neuropathy typically demonstrate axonal atrophy, demyelination, axonal degeneration with regeneration, and microangiopathy (Biessels et al., 2014; Malik et al., 1989, 2005; Thomas et al., 1996). We have previously shown that sural nerve endoneurial capillary density was increased and luminal area was decreased in subjects with impaired glucose tolerance (IGT) who progressed to type 2 diabetes (T2DM) (Thrainsdottir, Malik, & Dahlin, 2003). We have also recently shown that subjects with IGT may already have a significant small fiber neuropathy (Asghar et al., 2014) and that subjects with IGT who develop T2DM have a greater degree of small fiber pathology (Azmi et al., 2015).

Recently, an increasing body of evidence suggests that impaired macroautophagy (named autophagy), which is responsible for recycling of damaged organelles and misfolded proteins in conditions of cellular stress, may contribute to the chronic complications of diabetes (Sridhar, Botbol, Macian, & Cuervo, 2012; Varga et al., 2015; Wong & Cuervo, 2010). Thus, experimental data suggests that autophagic turnover may help to maintain healthy mitochondria and energy‐derived metabolic processes, limiting cellular death pathways, oxidative stress, and protein aggregation in diabetic neuropathy (Yerra, Gundu, Bachewal, & Kumar, 2016). Indeed, we have recently reported a more severe loss of large myelinated nerve fibers and increased unmyelinated fiber regeneration with a significant increase in autophagy structures in the posterior interosseous nerve of patients with type 1 compared to type 2 diabetes (Osman, Dahlin, Thomsen, & Mohseni, 2015).

We have had the unique opportunity to undertake detailed pathological quantification of nerve fibers and endoneurial capillaries in subjects with normal glucose tolerance (NGT), IGT, and T2DM at baseline and after 11 years of follow‐up. The aims of the current project were to assess whether the presence of autophagy‐related structures was associated with sural nerve fiber pathology in subjects with IGT and T2DM, and to investigate if endoneurial capillary pathology could predict the development of T2DM and neuropathy.

## 2 MATERIALS AND METHODS

The study protocol was approved by the Ethical Review Board at Lund University, Sweden (LU 275/90; LU 504‐03). Informed consent was obtained from all participants. The person who performed the electrophysiology examination was blinded to the subjects’ glucose tolerance, and all qualitative and quantitative microscopic evaluations were performed on coded samples.

We have previously reported some morphological and biochemical data from the baseline study (Sundkvist et al., 2000; Thrainsdottir et al., 2003, 2009). We have now undertaken detailed quantification of myelinated and unmyelinated nerve fibers and endoneurial vessels in relation to autophagy structures in the sural nerve at baseline and at follow‐up in the contralateral sural nerve 11 years after the initial sural nerve biopsy. Electron microscopy and morphometric analysis was undertaken to quantify nerve fiber pathology, endoneurial capillary pathology, and autophagy.

### 2.1 Subjects with NGT, IGT, and T2DM

Each group of subjects (males) was studied in 1991–1992 (i.e., baseline; Thrainsdottir et al., 2003; Sundkvist et al., 2000; Thrainsdottir et al., 2009) and followed‐up (i.e., follow‐up) in 2004. At baseline, subjects with NGT (*n* = 10), IGT (*n* = 10), and T2DM (*n* = 10) were randomly included from a larger population‐based health‐screening program initiated between 1975 and 1979 in Malmö, Sweden (Eriksson et al., 1994). Three subjects with NGT and 10 subjects with T2DM, of whom one was NGT and six were IGT at baseline were reassessed.

### 2.2 Electrophysiology

Sural nerve electrophysiology was performed at baseline and at follow‐up in accordance with the previously described electrophysiological technique (Sundkvist et al., 2000; Thrainsdottir et al., 2003, 2009).

### 2.3 Sural nerve biopsy

A whole sural nerve biopsy was taken by the same surgeon (LD) using the same previously described technique (Dahlin, Eriksson, & Sundkvist, 1997) with a median follow‐up of 11.0 years between the first and second biopsy (mean 11.2 years; 25th–75th percentiles 10.5–12 years; range 10–12 years).

### 2.4 Electron microscopy

Sural nerve biopsies were fixed in cacodylate‐buffered glutaraldehyde (25 g/L) and osmium tetroxide (10 g/L), and dehydrated in ethanol. The tissues were then infiltrated with propylene oxide, embedded in epoxy resin, sectioned (60 nm) using a ultratome (Leica Microsystems, Germany), and stained with 10% uranyl acetate for 20 min followed by 0.4% lead citrate for 3 min. A JEOL JEM‐1200EX transmission electron microscope (JEOL, Peabody, MA, USA) was used to acquire images for qualitative and quantitative analysis. At baseline, myelinated nerve fiber density was quantified in all subjects, but detailed morphometric analysis of nerve fibers excluded three NGT and one subject with T2DM, and for endoneurial capillaries two subjects with NGT and one subject with IGT, due to inadequate fixation for electron microscopy.

### 2.5 Myelinated fibers

Micrographs of one whole fascicle were prepared (×1,200 magnification) and the density of myelinated fibers (number of fibers/mm^2^) was calculated by dividing the total number of myelinated fibers by the fascicular area. The diameters of all myelinated fibers (axon + myelin) in that fascicle were measured using ImageJ (NIH; RRID: SCR_003070, version 1.47q; http://imagej.nih.gov/ij/). Fifty myelinated fibers were randomly selected and their axon diameter was quantified to calculate the *g*‐ratio.

### 2.6 Unmyelinated fibers

Twenty‐five micrographs (×15,000 magnification) were randomly prepared for each sample by starting in one corner of the fascicle and then every third microscopic field was photographed, while the fascicle was systematically scanned. For unmyelinated axon density, all axons in each picture were included, and density was calculated by dividing the number of axons by the area of each micrograph. For quantifying the axon diameter, only round and oval‐shaped axons were analyzed.

### 2.7 Endoneurial capillaries

All endoneurial vessels in a fascicle were photographed at ×2,500 to ×40,000. Vessels located in the perineurium and those with a major to minor axis ratio greater than 3:1 were excluded. Quantification was undertaken using our previously established morphometric techniques (Malik et al., 1989). The lumen area was calculated by tracing the inner endothelial cell border. The endothelial cell area was calculated by tracing the outer border of the endothelial cells and subtracting the lumen area. The basement membrane area was calculated by following the outer border of the basement membrane and subtracting the endothelial cell and lumen area; the total diffusion area excluded only lumen area. The vessel area was measured as the area within the outer basement membrane border. The fraction (%) of lumen area, endothelial cell area, and basement membrane area of the vessel was then determined. The number of endothelial cells/capillary was counted by counting the intercellular junctions. The number of endoneurial capillaries in the fascicle was counted and the area of the fascicle was measured to derive the endoneurial capillary density.

### 2.8 Autophagy structures

The density and cellular distribution of autophagy‐related structures, i.e., lysosomes, phagophores (pre‐autophagosomal membrane), autophagosomes (elongation of phagophore to double‐membraned vesicle), and autolysosome‐like structures (vesicles with one limiting membrane containing cytoplasmic compartment and organelles at various stages of degradation) (Klionsky et al., 2016) were counted in each micrograph and presented as number/mm^2^ as well as proportion of their cellular distribution (%).

### 2.9 Statistical analyses

The median values (25th–75th percentiles) are presented unless otherwise stated. The following statistical analysis were performed: Kruskal–Wallis rank sum tests followed by two‐tailed Mann–Whitney *U* tests for independent measures when comparing the three groups at baseline; two‐tailed Mann–Whitney *U* tests for independent measures when comparing the two groups at follow‐up; and the Wilcoxon signed‐rank test for matched participants, when comparing the first and second biopsies. Spearman's rank correlation was used to test association between variables. A *p* value ≤ .05 was considered as statistically significant. Statistical analyses were performed using SPSS (SPSS: RRID: SCR_002865; IBM^®^ SPSS^®^ Inc., Chicago, IL, USA; version 23.0; 2015).

## 3 RESULTS

The age, body mass index, HbA1c, and electrophysiological and morphological data for the sural nerve are presented in Table 1. At follow‐up, one subject with NGT and six subjects with IGT had converted to T2DM.

**Table 1:** Clinical and electrophysiological data obtained at baseline and follow‐up of subjects with NGT, IGT, and T2DM

Parameters	NGT	NGT	IGT	T2DM	T2DM
	Baseline *n *= 10	Follow‐up *n* = 3	Baseline *n* = 10	Baseline *n* = 10	Follow‐up *n* = 10
Age	64.0 (63.0–64.0)	74.0 [74.0–75.0]	64.0 (63.8–65.3)	62.0 (62.0–64.0)	75.5 (74.7–76.0) *p *≤* *.01^c^
BMI	26.1 (24.7–28.0)	28.8 [25.8–29.2]	26.7 (25.3–29.5)	27.2 (26.1–28.7)	26.3 (24.4–28.9)
HbA1c %mmol/mol	4.6% (4.2–5.1) 27.0 (22.0–32.0)	4.4 [4.3–4.5] 25.0 [23.0–26.0]	5.0 (4.5–5.7) 31.0 (26.0–117.0)	7.5 (6.7– 8.6) 58.0 (50.0–70.0) *p *≤* *.001^a^	5.3 (4.5–7.6) 34.0 (26.0–60.0)
SNCV (m/s)	44.0 (42.0–46.0)	44.0 [37.0–49.0]	47.0 (43.8–47.0)	41.0 (38.3–45.0) *p *≤* *.05^b^	40.0 (24.8–43.3) *p *=* *.03^c^
SNAP (μV)	9.0 (5.3–17.0)	9.0 [4.0–10.0]	11.3 (3.9–15.4)	3.7 (2.2–6.4) *p *≤* *.05^a^	2.5 (0.8–6.3) *p *≤* *.001^d^ *p *=* *.005^c^

SNCV, sural nerve conduction velocity; SNAP, sural nerve amplitude; NGT, normal glucose tolerance; IGT, impaired glucose tolerance; T2DM, Type 2 diabetes; BMI, body mass index.

Values are median (25th–75th percentiles) or when few numbers median [min–max].

Kruskal–Wallis with post hoc Mann–Whitney at baseline: ^a^Versus NGT and IGT; ^b^Versus IGT; Wilcoxon signed‐rank test: ^c^Versus baseline. ^d^Versus NGT and IGT. At follow‐up, three NGT subjects remained NGT. One subject with NGT and six subjects with IGT who had converted to T2DM as well as three of the patients with T2DM at baseline were recruited for the follow‐up study.

### 3.1 Baseline (NGT vs. IGT vs. T2DM)

#### 3.1.1 Electrophysiology

There was a significant difference in sural nerve conduction velocity (Kruskal–Wallis, *p *=* *.04) and amplitude (Kruskal–Wallis, *p *=* *.034) between the three groups. Post hoc analyses (Mann–Whitney) showed the lowest nerve conduction velocity in subjects with T2DM compared to IGT and significantly lower amplitude in T2DM compared to IGT and NGT (Table 1).

#### 3.1.2 Autophagy structures

**Figure 1:**
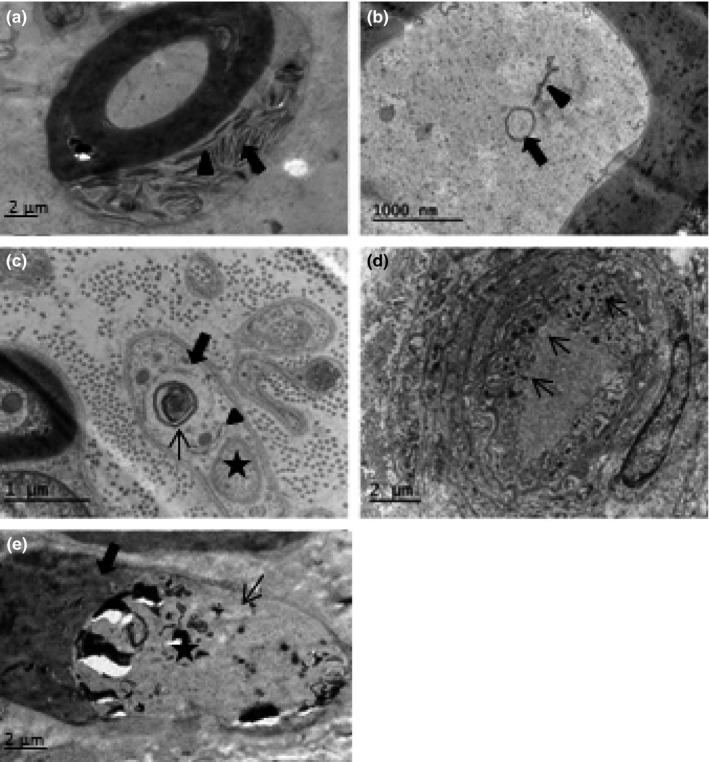
Electron micrograph showing (a) zebra bodies (arrow) and π‐granules (arrowhead), (b) double‐membraned autophagosome (arrow) and autophagophore (arrowhead) in a myelinated axon, (c) autolysosomes (thin arrow), the outer membrane of autophagosome (thick arrow), and autophagophore (arrowhead) in a Schwann cell of an unmyelinated axon (asterisk), (d) lysosomes in endothelial cells (arrows), and (e) a segmentally demyelinated axons; thick arrow shows myelin and thin arrow indicates unmyelinated segment of an axon (asterisk)

Glycogen bodies, fat droplets, multilamellar zebra bodies, and Reich granules (π granules; Figure 1a) were present in all samples. Autophagy‐like structures occurred mostly in myelinated and unmyelinated axons, and less frequently in Schwann cells and endothelial cells or pericytes (Figure 1b–d). No statistically significant differences were found between the groups in the total number of autophagy‐related structures (Table 2).

**Table 2:** Cellular distribution of autophagy structures in sural nerves of subjects with NGT, IGT, and T2DM at baseline and follow‐up

	NGT %	NGT %	IGT %	T2DM %	T2DM %
	Baseline *n* = 7	Follow‐up *n* = 3	Baseline *n* = 10	Baseline *n* = 9	Follow‐up *n* = 9
Schwann cells	20	54	24	24	62
Myelinated axons	50	14	29	27	0.1 *p *=* *.008^a^
Unmyelinated axons	27	30	45	47	22
Other cells	3	2	3	2	16
Lysosomes	42	66	33	29	70

NGT, normal glucose tolerance; IGT, impaired glucose tolerance; T2DM, type 2 diabetes mellitus; other cells, fibroblasts and endothelial cells.

^a^ Versus baseline. At follow‐up, three NGT subjects remained NGT. One subject with NGT and six subjects with IGT who had converted to T2DM as well as three of the patients with T2DM at baseline were recruited for the follow‐up study. At baseline, identification of autophagy structures was not performed in three NGT and one subject with T2DM due to poor morphology of organelles and structures because of inadequate fixation for electron microscopy.

### 3.2 NGT

Segmental demyelination was observed only in two fibers (Figure 1e). The median myelinated nerve fiber density was 5,662/mm^2^ and the myelinated nerve fiber size frequency distribution was bimodal with peaks at 3–5 μm and 9–12 μm. The median myelinated nerve fiber diameter was 7.3 μm, and axon diameter was 3.9 μm with a *g*‐ratio of 0.58 (Table 3). The median unmyelinated axon density was 31,309/mm^2^, and the median axon diameter was 1.1 μm (Table 3). Morphometric data for endoneurial blood vessels is provided in Table 3. Autophagic structures were found primarily in the myelinated fiber axons and to a lesser extent in unmyelinated axons and Schwann cells, with very few in other cells (Table 2).

**Table 3:** Morphometric data obtained from human sural nerves at baseline and follow‐up of subjects with NGT, IGT and T2DM

Parameters	NGT	NGT	IGT	T2DM	T2DM
	Baseline *n* = 10	Follow‐up *n* = 3	Baseline *n* = 10	Baseline *n* = 10	Follow‐up *n* = 10
Myelinated fiber diameter (μm)	7.3 (6.0–8.2)	5.7 [5.5–7.3]	6.0 (5.5–6.8)	6.3 (5.7–6.6)	6.0 (5.0–6.9)
Myelinated axon diameter (μm)	3.9 (3.6–4.6)	3.4 [3.1–3.8]	3.5 (3.2–3.8) *p *<* *.03^a^	3.4 (3.3–3.7) *p *<* *.03^a^	3.6 (3.1–4.0)
Unmyelinated axon diameter (μm)	1.1 (1.0–1.2)	1.0 [0.96–1.2]	1.1 (1.1–1.2)	1.1 (1.1–1.2)	0.98 (0.81–1.1) *p = *.038^b^
*g*‐Ratio	0.58 (0.55–0.63)	0.57 [0.53–0.64]	0.60 (0.56–0.61)	0.58 (0.57–0.60)	0.63 (0.57–0.66)
Myelinated fiber density (no./mm^2^)	5,662 (5,237–6,593)	5,125 [4,172–6,656]	6,384 (5,105–8,339)	6,439 (4,512–9,767)	2,839 (2,125–3,962) *p *=* *.005^b^ *p *=* *.028^a^
Unmyelinated axon density (no./mm^2^)	31,309 (22,708–38,190)	33,373 [19,712–36,126]	25,976 (24,686–35,793)	22,708 (20,127–33,201)	27,180 (20,557–34,922)
Autophagy density (no./mm^2^ in the nerve)	7,225 (2,064–9,633)	4,817 [3,441–6,881]	4,645 (2,666–6,193)	6,537 (5,160–11,182)	4,473 [2,408–7,483]
Endoneurial capillary density (no./mm^2^)	49.0 (34.3–54.9)	43.9 (32.9–58.6)	54.9 (21.5–77.8)	61.2 (52.8–68.3)	44.7 (31.0–74.6) *n* = 9
Lumen area (μm^2^)	19.7 (8.3–23.7)	12.8 [8.3–28.0]	5.5 (1.7–21.0)	11.6 (1.9–19.4)	8.5 (5.6–24.5)
Lumen area (%)	10.2 (4.7–14.7)	11.1 [8.0–13.0]	6.9 (1.6–11.0)	5.0 (1.1–9.8)	5.5 (1.4–11.8)
EC area (μm^2^)	28.9 (19.5–42.5)	50.3 [32.9–70.9]	28.2 (16.6–38.4)	25.5 (22.9–39.0)	40.9 (34.5–62.9) *p *≤* *.05^b^
EC area (%)	14.2 (7.5–23.4)	17.0 [11.8–26.1]	23.6 (20.7–27.7)	17.6 (11.3–29.3)	21.6 (16.9–23.3)
EC number	4.0 (4.0–4.0)	4.0 [3.5–5.0]	5.5 (4.4–6.3)	4.0 (4.0–4.5)	5.0 (4.8–5.1)
BM area (μm^2^)	124.0 (88.7–155.3)	114.5 [101.5–234.8]	66.4 (48.3–92.5) *p *<* *.03^a^	142.4 (60.1–164.2)	133.1 (73.2–161.3)
BM area (%)	66.5 (60.7–78.8)	74.4 [63.3–76.4]	68.5 (60.9–73.3)	70.0 (63.0–79.5)	69.9 (64.0–78.2)
Total diffusion barrier (μm^2^)	144.5 (110.2–164.9)	133.9 [94.7–267.4]	90.4 (62.6–133.3) *p *<* *.08^a^	152.4 (77.6–177.0)	174.0 (114.8–214.6) *p *<* *.02^b^

IGT, impaired glucose tolerance; *n*, number of patients; BM, basement membrane; EC, endothelial cell; TDB, total diffusion barrier; NGT, normal glucose tolerance.

Data are presented as median and (25th–75th percentiles) or when few numbers as [min–max].

Detailed nerve fiber morphometric analysis could not be performed at baseline due to poor fixation in one subject with NGT and one with T2DM. Blood vessel analysis at baseline was undertaken in eight subjects with NGT, nine subjects with IGT and 10 subjects with T2DM. At follow‐up, one patient with T2DM was excluded due to poor quality of the tissue. One subject with NGT and six subjects with IGT who had converted to T2DM as well as three of the patients with T2DM at baseline were recruited for the follow‐up study.

^a^Versus NGT (Kruskal–Wallis with post hoc analyses using Mann–Whitney at baseline and Mann–Whitney for the follow‐up).

^b^Versus baseline (Wilcoxon signed‐rank test).

### 3.3 IGT

The median myelinated nerve fiber density was 6,384/mm^2^ and the myelinated nerve fiber size frequency distribution was bimodal with peaks at 3–5 μm and 9–10 μm. The median myelinated nerve fiber diameter was 6.0 μm and comparable to NGT, while the axon diameter was significantly smaller than in subjects with NGT (*p *<* *.03) (Table 3). The median unmyelinated axon density was 25,976/mm^2^, and the median axon diameter was 1.1 μm and did not differ from subjects with NGT (Table 3). Endoneurial capillary density, lumen area, endothelial cell area, and number were comparable, but basement membrane area (*p *<* *.03) and total diffusion barrier area (*p *<* *.03) were significantly lower than in subjects with NGT (Table 3).

### 3.4 T2DM

**Figure 2:**
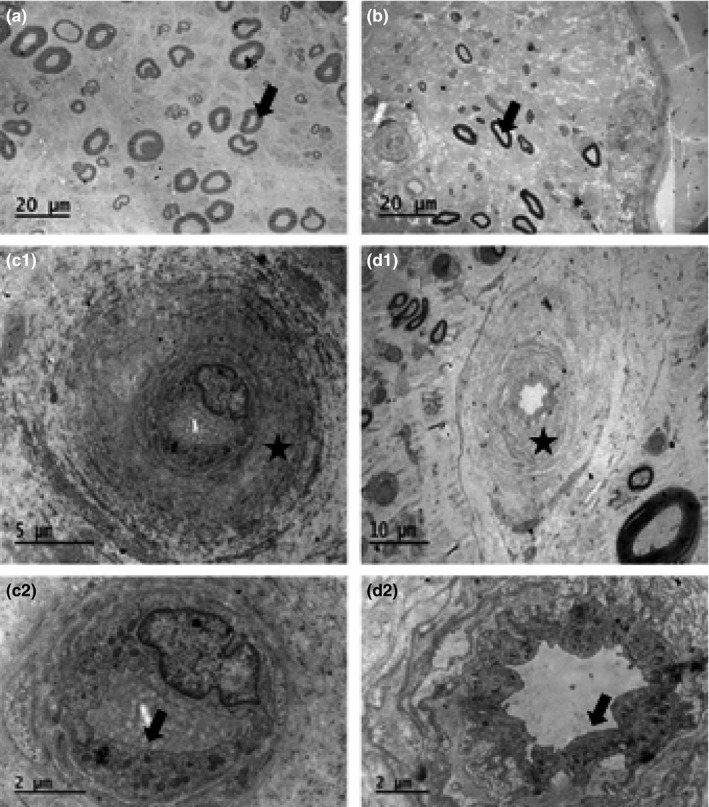
Sural nerve of a patient with type 2 diabetes at baseline (a), who was severely affected by neuropathy at follow‐up (b; arrows indicate myelinated axons). The microvessels of a control subject (c1; higher magnification in c2) and a patient with type 2 diabetes (d1; higher magnification in d2) are shown. Asterisks indicate basement membrane (c1 and d1) and arrows indicate endothelium (c2 and d2)

The median myelinated nerve fiber density was 6,439/mm^2^ and did not differ from NGT or IGT. The myelinated nerve fiber size frequency was bimodal with peaks at 3–4 μm and 8–10 μm. The median myelinated fiber diameter was 6.3 μm (Figure 2a), but the axon diameter was 3.4 μm and was significantly smaller than in subjects with NGT (*p *<* *.03) (Table 3). The median unmyelinated axon density was 22,708/mm^2^ and the median axon diameter was 1.1 μm and did not differ from subjects with NGT or IGT (Table 3). Endoneurial capillary density, lumen area, endothelial cell area, basement membrane area, and total diffusion barrier area did not differ significantly from subjects with NGT or IGT (Table 3; Figure 2c1–d2). Myelinated fiber density correlated negatively with EC area (*r* = −.44; *p *=* *.03).

### 3.5 Differences over time

#### 3.5.1 Patients with T2DM

In the 10 patients with T2DM at follow‐up (baseline NGT = 1, IGT = 6, and T2DM = 3), there was a significant reduction in amplitude (*p *=* *.005) and conduction velocity (*p *=* *.03; Table 1). Myelinated nerve fiber density was lower at follow‐up (*p *=* *.005; Figure 2a,b), but there were no changes in myelinated nerve fiber diameter, axon diameter, or *g*‐ratio (Table 3). Unmyelinated axon density remained unchanged but axons had smaller diameter versus baseline (*p* = .038). Endothelial cell area was significantly higher at follow‐up (*p = *≤.05; Table 3). Autophagy structures were absent in the myelinated axons at follow‐up (*p *=* *.008; Table 2) compared to baseline.

### 3.6 Subjects who developed T2DM

Subjects who developed T2DM had a smaller endoneurial capillary lumen area (*p* < .02) and larger percentage of endothelial cells (*p* < .04), and slightly higher *g*‐ratio (*p* = .056) at baseline versus those who remained NGT (Table 4). The seven subjects (1 NGT + 6 IGT), who converted to T2DM, demonstrated a significant reduction in myelinated nerve fiber density (*p* < .02) and sural nerve amplitude (*p* < .02) and an increase in myelinated axon diameter (*p* < .03), indicating a loss of mostly smaller myelinated axons, but no change in unmyelinated fibers versus baseline (Table 4). Basement membrane and total diffusion barrier was slightly increased in T2DM (*p *=* *.063; Table 4).

**Table 4:** Data obtained from NGT patients who remained NGT at follow‐up (NGT), and those from one subject with NGT and six subjects with IGT at baseline who converted to T2DM at follow‐up

Parameters	NGT	NGT + IGT	T2DM (baseline NGT + IGT)
	Baseline *n* = 3	Baseline *n* = 7	Follow‐up *n* = 7
Lumen area (μm^2^)	22.8 (22.1–30.2)	5.5 (1.7–20.6) *p *<* *.02^a^	9.5 (5.6–27.3)
Lumen area (%)	14.7 (13.7–17.8)	6.8 (1.1–9.6) *p *<* *.02^a^	7.0 (1.4–12.5)
EC area (%)	14.2 (7.5–20.6)	23.6 (21.5–24.3) *p *<* *.04^a^	21.7 (17.0–24.4)
*g*‐Ratio	0.53 (0.51–0.55) *n* = 2	0.60 (0.56–0.63) *p = *.056^a^	0.64 (0.57–0.68)
SNAP (μV)	11.8 (10.0–17.0)	12.6 (6.0–15.1)	4.0 (1.0–7.0) *p < *.02^b^
Myelinated axon diameter (μm)	3.8 (3.7–3.9)	3.6 (3.2–3.6)	3.8 (3.5–4.1) *p < *.03^b^
Myelinated fiber density (no./mm^2^)	6,121 (5,649–6,593)	6,070 (4,930–6,389)	2,973 (2,163–4,808) *p < *.02^b^
BM area (μm^2^)	164.7 (120.5–166.8)	123.9 (84.3–145.3)	177.7 (114.6–236.0) *p = *.063^b^
Total diffusion barrier (μm^2^)	134.6 (102–153.7)	100.9 (82.5–142.5)	141.4 (102.8–227.3) *p = *.063^b^

NGT, normal glucose tolerance; IGT, impaired glucose tolerance; T2DM, type 2 diabetes; *n*, number of patients; EC, endothelial cell; SNAP, sural nerve amplitude; TDB, total diffusion barrier; BM, basement membrane.

Data are presented as median (25th–75th percentiles); data for the three healthy subjects at baseline are median (min–max).

^a^ Versus NGT.

^b^ Versus NGT + IGT at baseline. One subject with NGT and six subjects with IGT at baseline had converted to T2DM at follow‐up.

## 4 DISCUSSION

In this study, we observed lower sural nerve amplitude in subjects with T2DM while smaller myelinated axon diameter occurred in T2DM and IGT patients at baseline. The closer analyses of the baseline data revealed that the individuals with NGT or IGT who converted to T2DM 11 years later (follow‐up) had smaller endoneurial capillary and higher *g*‐ratio versus those who remained NGT. These factors may presage the development of T2DM and neuropathy. At follow‐up, signs of severe neuropathy and microangiopathy were observed in patients with T2DM. The novel finding of the current study was the absence of autophagy structure in myelinated axons at follow‐up.

Autophagy is a regulated process of degradation and recycling of cellular constituents responsible for organelle turnover (Klionsky & Emr, 2000) which has been shown to be disrupted in a range of central neurodegenerative conditions and myopathies (Kiriyama & Nochi, 2015; Maday, 2016). Indeed, we have recently demonstrated a significant increase in the density of autophagy‐related structures in myelinated and unmyelinated axons and particularly Schwann cells of sural nerves from patients with chronic idiopathic axonal neuropathy and inflammatory neuropathy (Samuelsson et al., 2016). However, in the present study we show no difference in the density of autophagy structures in subjects with NGT, IGT, and T2DM at baseline, which is in agreement with our recent study showing no difference in autophagy structures in the posterior interosseous nerve of patients with T2DM (Osman et al., 2015). Furthermore, in the current study, we demonstrate a significant decrease in autophagy structures in the myelinated axons of patients with T2DM at follow‐up, despite increasing severity of neuropathy evidenced by worsening neurophysiology and nerve fiber pathology. Although not significant, the proportion of autophagy structures had also decreased in unmyelinated axons while it was increased in Schwann cells at follow‐up. Indeed, it has recently been reported that Schwann cells use autophagy as a degradation process (Gomez‐Sanchez et al., 2015; Jang et al., 2016). Obviously, the autophagy pathway is affected in both axons and Schwann cells in long‐term diabetes. Generally, accumulation of autophagy structures in the cell, i.e., Schwann cells in the current study, is taken as a sign of inhibition of autophagy pathway but this can only be determined by assessing autophagy flux (Klionsky et al., 2016), which can only be quantified in fresh nerve tissue which was not available in the present study.

This longitudinal study has allowed us to explore how alterations in endoneurial capillaries relate to the development and progression of neuropathy over 11 years. Patients with T2DM show a significant progression of endoneurial microangiopathy and neuropathy with an increased risk of myocardial infarction and mortality (Hanefeld et al., 1996). We have previously shown a significant microangiopathy evidenced by basement membrane thickening, reduction in luminal size, and endothelial cell hyperplasia in patients with initially minimal but progressive diabetic neuropathy (Malik et al., 2005). In patients with T2DM, we show basement membrane thickening and endothelial cell hyperplasia and hypertrophy as also reported by others (Giannini & Dyck, 1995; Malik et al., 1989, 2005; Thrainsdottir et al., 2003; Yasuda & Dyck, 1987) without a reduction in luminal area which agrees with some (Giannini & Dyck, 1995), but not other (Dyck et al., 1985; Malik et al., 2005) studies. In the present study, in patients with T2DM, there were no evidence of microangiopathy at baseline but it developed over 11 years as a consequence of hyperglycemia and other cardiovascular risk factors (Calabek, Callaghan, & Feldman, 2014; Callaghan, Cheng, Stables, Smith, & Feldman, 2012; Callaghan et al., 2012). Interestingly, subjects who developed diabetes at follow‐up had a smaller endoneurial capillary lumen at baseline, which confirms our previous smaller study showing a smaller endoneurial capillary luminal area in subjects with a deterioration in glucose tolerance (Thrainsdottir et al., 2003).

In relation to neuropathy in T2DM, axonal degeneration, regeneration, demyelination, and remyelination have been reported in some (Hur, Sullivan, Callaghan, Pop‐Busui, & Feldman, 2013), but not all studies (Sundkvist et al., 2000), reflecting differences in disease duration, metabolic control and the sensitivity of the methods employed to evaluate pathology (e.g., light microscopy versus electron microscopy). In the present study, at baseline sural nerve amplitude and conduction velocity were reduced with no difference in myelinated fiber density, but at follow‐up, all three parameters were significantly reduced which may be related to long duration of poorer glycemic control.

In subjects with IGT, the main pathology was a reduction in myelinated axon diameter at baseline. A peripheral axonal neuropathy affecting small fibers in subjects with IGT has been described in some, but not other (Dyck et al., 2012; Eriksson et al., 1994), studies. We have previously demonstrated a significant small fiber neuropathy in skin biopsies and using corneal confocal microscopy in subjects with IGT (Asghar et al., 2014), particularly those who develop T2DM (Azmi et al., 2015). In the current study, while sural nerve amplitude and myelinated fiber density were decreased in the subjects who developed T2DM at follow‐up, there was no change in unmyelinated fiber density, suggesting a lack of pathology in these fibers in the proximal sural nerve. We conclude that ultrastructural evidence of autophagy was observed in human sural nerves of subjects with NGT, IGT, or T2DM at baseline and after 11 years of follow‐up. Further studies are warranted to understand the role of autophagy in diabetic neuropathy. Small endoneurial capillary luminal size may presage the development of T2DM and neuropathy.

## CONFLICT OF INTEREST

The authors have declared that no conflicts of interest exist.
